# Reliability of a Fully Automated Interpretation of ***γ***-H2AX Foci in Lymphocytes of Moderately Trained Subjects under Resting Conditions

**DOI:** 10.1155/2014/478324

**Published:** 2014-07-24

**Authors:** Juliane Heydenreich, Christoph Otto, Frank Mayer, Anja Carlsohn

**Affiliations:** ^1^Sports Medicine and Sports Orthopaedics, University Outpatient Clinic, University of Potsdam, 14469 Potsdam, Germany; ^2^ZIEL-Research Centre for Nutrition and Food Sciences, Munich Technical University, 85354 Freising-Weihenstephan, Germany; ^3^Institute of Health Science, Schwäbisch Gmünd University of Education, 73529 Schwäbisch Gmünd, Germany

## Abstract

*Background*. Analysis of *γ*-H2AX foci is a promising approach to evaluate exercise-induced DNA damage. However, baseline levels and day-to-day variability of *γ*-H2AX foci have not been investigated in healthy subjects at rest. *Methods*. Blood was taken from eight moderately trained healthy males (29 ± 3 yrs, 1.84 ± 0.03 m, and 85 ± 6 kg) at two separate days (M1/M2) after 24-hour exercise cessation. Number of *γ*-H2AX foci per 100 lymphocytes (N), number of foci per affected lymphocyte (NAL), percentage of affected lymphocytes (PAL), and diameter (D) of *γ*-H2AX foci were analyzed (mean ± SD). Differences between M1 and M2 were analyzed using paired *t*-tests (*α* = 0.05). Day-to-day variability was evaluated by calculating the coefficients of variation (CV%), bias, and limits of agreement (LoA). *Results*. There were no statistically significant differences between M1 (N: 7.6 ± 4.4, NAL: 1.2 ± 0.2, PAL: 5.9 ± 2.6%, and D: 0.63 ± 0.07) and M2 (N: 8.4 ± 4.6, NAL: 1.3 ± 0.1, PAL: 6.9 ± 4.2%, and D: 0.66 ± 0.06). CV was calculated to be 98.5% (N), 88.9% (PAL), 11.3% (NAL), and 8.0% (D). Bias (LoA) was 0.75 (−15.2/13.7), −0.02 (−0.36/0.33), −1.0 (−11.9/9.9), and −0.04 (−0.16/0.09), respectively. *Conclusions*. Background level in healthy subjects is approximately 0.07 to 0.09 *γ*-H2AX foci/cell. NAL and D are reliable measures.

## 1. Introduction

Exercise has been shown to induce an increased formation of reactive oxygen species (ROS) such as superoxide, hydroxyl radicals, or hydrogen peroxide [1, 2]. Sources of exercise-induced ROS production are the mitochondrial electron transport chain [3–5], the xanthine oxidase pathway [6], activated neutrophiles [6], and membrane-bound NAD(P)H oxidases of the skeletal muscle [7, 8]. Excess ROS generation may damage cell components such as lipids, proteins, and nucleic acids [9]. Exercise-induced DNA damage has been observed following acute bouts of exhaustive, prolonged, or eccentric exercises in humans [10–12]. Here, DNA double-strand breaks are the most harmful lesions and may occur due to different treatments [13].

Although acute bouts of unaccustomed exercises may induce DNA damage, chronic exercises as well as adequate antioxidant intake may alleviate oxidative damage [14–16]. For example, Miyazaki et al. have shown that twelve weeks of high-intensity endurance training in previously untrained men alleviates oxidative stress induced by an acute session of exercise [15].

Regarding antioxidant intake, Harms-Ringdahl et al. have shown that exercise-induced increase of 8-oxodG, as a marker of oxidative stress, is reduced after five weeks of daily tomato juice consumption containing approximately 15 mg lycopene per serving [17]. A daily intake of 1200 mg vitamin E for 14 days was capable of reducing exercise-induced DNA strand breakage in white blood cells of moderately trained subjects [18]. Following an acute ultraendurance event, a significant inverse correlation between oxygen radical absorbance capacity (ORAC) of the blood and indices of oxidative DNA damage was found. In the same study, a significant increase in blood antioxidants immediately after the race was observed [19].

Thus, methods that reliably measure DNA damage, despite the noise of day-to-day variability in antioxidant intake and exercise intensity, kind, and duration, are required to assess the effects of acute bouts of exercises in trained subjects.

The analysis of *γ*-H2AX foci is a promising marker of DNA double-strand breaks* in vitro* and* in vivo* [20]. The histone protein H2AX serves as key factor in the repair process of DNA damage, which targets damaged DNA and recruits other proteins of the DNA repair machinery [21]. As an exception among H2A proteins H2AX is phosphorylated on the serine 139 residue in the presence of damaged DNA. The phosphorylated H2AX (*γ*-H2AX) may be visualized using fluorescence labeled antibodies. This allows the counting of cells with damaged DNA, to calculate the number of foci per 100 cells, and to measure the diameter of *γ*-H2AX foci. Analysis of *γ*-H2AX foci has been shown to accurately measure DNA double-strand breaks [22]. Visual interpretation of fluorescent foci patterns and counting *γ*-H2AX foci with the naked eye is time consuming and has some limitations, such as investigator-related artefacts [23]. Recently, a fully automated device has been developed and has been shown to highly correlate with visually analyzed foci counts [23].

As the analysis of *γ*-H2AX foci is a sensitive marker of DNA damage and has been proven to detect DNA double-strand breaks at low levels of DNA damage [24], it promises to be a valuable tool to evaluate exercise-induced DNA damage. For well-trained athletes, however, regular exercise training seems to evoke adaptative responses capable of reducing oxidative damage [3, 25–27]. Therefore, the day-to-day variability of *γ*-H2AX foci in trained subjects under resting conditions needs to be investigated before assessing the effects of a given exercise bout in athletes.

Therefore, the purpose of this study was to analyze the reliability of *γ*-H2AX foci in lymphocytes of moderately trained subjects under resting conditions.

## 2. Methods

### 2.1. Participants

Twelve moderately trained healthy males were recruited to participate in the study. Inclusion criteria comprised an age between 25 and 40 years and a body mass index between 18 and 25 kg/m². Participants were moderately active with one to three exercise sessions per week. Females, smokers, and volunteers with acute or chronic diseases were excluded from the study. Changes of dietary behaviour or of exercise loads, alcohol intake, supplement use, or any medication during the study period served as exclusion criteria. Subjects who performed exercise training within 24 hours before the measurements were excluded from the analysis (*N* = 4).

Finally, eight subjects were included into the study and the data analysis (29.3 ± 3.2 yrs, 184 ± 3 cm, and 84.7 ± 6.1 kg). All subjects gave written informed consent before participating in the study. The study was approved by the institutional scientific examination board.

### 2.2. Study Design

The study was conducted in a test-retest design, repeating the same measurements of day 1 (M1) after seven days (M2) under standardized conditions. Before the first measurement, participants underwent a clinical examination, where inclusion and exclusion criteria were verified by means of a checklist. After refraining from exercise training for 24 hours, venous blood samples were taken. During the whole duration of the study, participants were asked to avoid strenuous physical activity. A physical activity record that included duration, kind, and ratings of perceived exertion of exercise was handed out to verify that subjects followed the instructions on physical activity.

### 2.3. Blood Analysis

Venous blood samples at M1 and M2 were taken from the brachial vein between 7 and 9 a.m. to minimize the effect of physical activity before the blood sample collection. Commercial tubes coated with EDTA to prevent blood clotting were used to collect 2 × 2.7 mL of whole blood. Immediately after blood draw, samples were stored at 4°C. One blood sample was used to analyze blood parameters (hematocrit [%], haemoglobin [g/dL], amount of lymphocytes [%], and number of red blood cells [10^6^/mm³]) and concentration of creatine kinase [U/L].

The other blood sample was used to assess double-strand breaks (DSB) in lymphocytes. In the first step, isolated cells were fixed in a neutral buffered 4% formaldehyde solution (Merck, Darmstadt, Germany) for 15 minutes at room temperature. Afterwards, the samples were washed three times with PBS (Merck, Darmstadt, Germany). Cell membranes of the fixed cells were incubated for five minutes with Triton X100 (Merck, Darmstadt, Germany; 0.1% in PBS v/v), followed by other three washings with PBS. Afterwards, unspecific binding was blocked using a BSA/PBS buffer. In the third step, a primary antibody targeting *γ*-H2AX was added which was then bound to a secondary antibody conjugated with Alexa Fluor 488 (Invitrogen, Karlsruhe, Germany). To detect antibody-bound *γ*-H2AX, a fluorescent mounting medium (Dako Deutschland, Hamburg, Germany) was added. 4,6′-Diamidino-2-phenylindole (DAPI, Sigma-Aldrich) was used to dye all cells' nuclei.

To analyse *γ*-H2AX foci in lymphocytes and to count the cells an automated indirect immune fluorescence device was adopted (AKLIDES Nuk Human Lymphocyte Complete, Medipan, Dahlewitz, Germany). A pattern recognition algorithm of the automated fluorescence interpretation system AKLIDES (Medipan, Dahlewitz, Germany) was used for evaluation of *γ*-H2AX foci as described elsewhere [28, 29]. For determination of *γ*-H2AX foci a 60x magnification of the microscope objective was used.

The following parameters were assessed: (1) number of *γ*-H2AX foci per 100 cells, (2) number of cells with *γ*-H2AX foci per 100 cells, (3) mean number of *γ*-H2AX foci per cell with *γ*-H2AX foci, and (4) the mean diameter of *γ*-H2AX foci.

### 2.4. Standardization of Diet and Physical Activity

To alleviate the acute effects of diet and physical activity, both exercise training and antioxidant intake were standardized before blood sample collection. A standardized dietary protocol was used to record the nutritional intake 24 hours prior to both measurements. Written and oral instructions on how to record the diet were given. Descriptions of portion sizes and standard household measures were provided. Dietary records from the first measurement were handed out before the second measurement, and participants were asked to follow the same diet in the 24 hours before M2. Nutrient and antioxidant intake were analyzed using nutrition analysis software based on the German food database (Prodi NutriScience 5.7 expert, Hausach, Germany). All nutritional analyses were performed by the same experienced examiner (JH).

To avoid artefacts due to exercise, subjects were requested to refrain from any exercises and strenuous physical activity for at least 24 hours before the measurements. This request was given as oral and written instructions to the participants. To verify the subjects' compliance to physical activity instructions, participants were requested to record their exercise training as well as physical activity for transportation (i.e., bicycling to work) during the whole study period. Borg's scale was used to evaluate subjective ratings of perceived exertion (RPE) [30]. To confirm resting conditions and the absence of muscle cell damage, blood samples were analysed for creatine kinase (CK) concentration, which serves as a marker of muscle cell damage [31].

### 2.5. Statistical Analysis

All data are given in mean ± standard deviation (mean ± SD) and 95% confidence intervals where appropriate. Shapiro-Wilk test was used to test for normal data distribution. Paired* t*-tests were performed to assess differences in nutrient intake, physical activity, and blood parameters between M1 and M2 (*α* = 0.05). The Bland-Altman procedure was performed to assess reliability of the measurement procedure by calculating the bias (mean difference between M1 and M2) and the 95% limits of agreement [32, 33]. In addition, the coefficient of variation (CV%) was calculated with log-transformed data for the parameters of foci analysis. Statistical calculations were performed using JMP, Version 9 (SAS Institute Inc., Cary, NC).

## 3. Results

### 3.1. Physical Activity

Throughout the entire study period, subjects reported 24.2 ± 9.9 min of vigorous physical activity or exercise training per day with a subjective exertion of 13.4 ± 1.7 RPE. Subjects were compliant with the instruction to refrain from exercise the day before the measurements. There was no difference between M1 and M2 in exercise duration (M1: 1.3 ± 3.5 min/d; M2: 0 ± 0 min/d; *P* = 0.35) or perceived exertion (M1: 1.4 ± 3.9 RPE; M2: 0 ± 0 RPE; *P* = 0.33) during the 24 hours before the measurements. Blood concentration of creatine kinase did not differ between M1 (160.4 ± 85.7 U/L) and M2 (133.3 ± 73.5 U/L; *P* = 0.32).

### 3.2. Diet and Antioxidant Intake

There was no difference in energy intake 24 h before M1 (2407 ± 625 kcal/d) and M2 (2376 ± 517 kcal/d; *P* = 0.89). Recommendations for the daily intake of vitamins C and E and retinol equivalents were reached by all participants with no differences between M1 and M2. Mean intake of vitamin C was 137 ± 66 mg/d before M1 and 147 ± 54 mg/d before M2 (*P* = 0.68) and intake of vitamin E 16 ± 5 mg/d before M1 and 15 ± 7 mg/d before M2 (*P* = 0.57). For retinol equivalents, a mean intake of 3.2 ± 2.7 mg/d before M1 and 1.7 ± 1.0 mg/d before M2 (*P* = 0.78) was observed.

### 3.3. Analysis of *γ*-H2AX Foci

The total number of *γ*-H2AX foci detected per 100 lymphocytes was 7.6 ± 4.4 in M1 and 8.4 ± 4.6 in M2 (*P* = 0.79). The number of cells from 100 lymphocytes in which *γ*-H2AX foci were observed did not differ between M1 (5.9 ± 2.7) and M2 (6.9 ± 4.2; *P* = 0.58). In affected cells, a mean number of 1.2 ± 0.2 (M1) and 1.3 ± 0.1 (M2) *γ*-H2AX foci were found (*P* = 0.80). The mean diameter of *γ*-H2AX foci did not differ between the two measurements (M1: 0.63 ± 0.07 *μ*g, M2: 0.66 ± 0.06 *μ*g; *P* = 0.31). In Table 1 a detailed list of individual outcome measures for *γ*-H2AX foci is shown.

### 3.4. Reliability of the Outcome Measures

No measures of the *γ*-H2AX foci were significantly different between M1 and M2 (*P* > 0.05). The coefficient of variation (CV%), the bias, and the limits of agreement (LoA) for the four outcome variables are given in Table 2. CV of the particular outcome measures varied between 8.0% and 98.5%. The bias was generally close to zero except for the number of affected cells, where bias was −1.0. The 95% limits of agreement were narrow for the *γ*-H2AX foci diameter and the number of *γ*-H2AX foci per affected cell. However, wide limits of agreement were observed for the number of *γ*-H2AX foci per 100 lymphocytes and the percentage of affected cells.

## 4. Discussion

The analysis of *γ*-H2AX foci has been shown to accurately measure DNA double-strand breaks and to be sensitive even at low levels of DNA damage [22, 24]. As strenuous exercise is capable of inducing oxidative stress and DNA damage [12], the fully automated detection of *γ*-H2AX foci in human lymphocytes is a promising approach to evaluate exercise-induced DNA damage in athletes. However, until now, the day-to-day variation of this approach has not been investigated in healthy moderately active subjects.

The data of the current study suggest that the reliability of the analysis of *γ*-H2AX foci varies between the outcome measures. In general, there were no significant differences in the test-retest values of the four outcome parameters. However, the coefficient of variation was high for the total number of foci per 100 cells and the percentage of affected cells. For these two parameters, the bias was in an acceptable range and between zero and one, which is about 15% or less of the grand mean of the two repeatedly measured parameters. It remains unknown whether the variation between the test-retest situations is a methodological issue or reflects a real biological variation. For the COMET assay—a well-established method to quantify DNA damage in leucocytes—a high variation of values of DNA damage in healthy humans is known [34]. Additionally, Ismail et al. reported a high interindividual variability of *γ*-H2AX foci in human white blood cells in response to ionizing radiation, indicating that the number *γ*-H2AX produced during the early repair process of DNA double-strand breaks is different between subjects [22]. However, others have shown that the total number of *γ*-H2AX foci significantly correlates with the number of double-strand breaks [35].

In the present study large inter- and intraindividual differences in outcome values have been observed. For total number of foci per 100 cells individual values ranged from 2 to 15, whereas the range for percentage of affected cells was 2–14%, for number of foci per affected cell 1.0–1.6, and for diameter 0.53–0.76 *μ*m, respectively.

Little data are available about the total number of *γ*-H2AX foci in untreated human cells of healthy subjects.* In vitro*, Rothkamm and Löbrich found a mean of 0.05 foci per cell in a cell culture of human fibroblasts that was not treated with irradiation [36]. Approximately five *γ*-H2AX foci per 100 cells were regarded as the background level of *γ*-H2AX foci in untreated cells by the authors [36].* In vivo*, under normal conditions, *γ*-H2AX foci values less than one focus per cell are reported [37–39]. Unfortunately, most of the studies assessing *γ*-H2AX foci in lymphocytes are dealing with patients and/or older subjects of a mixed population with unequal gender. It remains unclear whether the observed background levels of *γ*-H2AX foci (0.07 to 0.08 *γ*-H2AX foci per cell) in moderately trained males are physiological. Rothkamm and Löbrich determined the variation in the levels of spontaneous *γ*-H2AX focus formation and found a mean of 0.04 and 0.06 foci per cell in different* in vitro* experiments [36]. Thus, the day-to-day variability of the number of *γ*-H2AX foci found by the authors in human cell lines is slightly lower but similar to the total number of foci found in the present study of human lymphocytes in healthy subjects under resting conditions.

Regarding reliability, a low systematic error with a bias very close to zero (−0.02 and −0.04) was found for both the number of *γ*-H2AX foci per affected cell and the mean diameter of the foci. Here, the coefficients of variation were within a typical range observed for day-to-day variability of biological outcome measures and below 12 CV%. However, it remains unknown whether the number of foci per affected cell or the diameter of the foci changes due to strenuous exercise.

Limitations of the study are the low number of subjects in the test-retest design and the missed opportunity to distinguish between biological variation and technical measurement error. Here, at each measurement, a second blood sample should have been collected immediately after the first blood sample to analyse the technical error.

The background level of 0.07 to 0.08 *γ*-H2AX foci per cell in the present study is comparable to values reported elsewhere and varies inter- and intraindividually under resting conditions. Future studies should be conducted, which include more subjects, to determine the resting level of *γ*-H2AX foci and accompanied parameters.

In conclusion, the analysis of the *γ*-H2AX focus diameter and the number of *γ*-H2AX foci per affected cell are reliable measures in healthy moderately active subjects. To analyse the effect of exercise on DNA damage the high day-to-day variability of total number of *γ*-H2AX foci needs to be considered. Whether exercise affects the prevalence of *γ*-H2AX in lymphocytes of healthy trained subjects needs to be investigated.

## Figures and Tables

**Table 1:** Individual values for the four parameters of the *γ*-H2AX foci analysis in human lymphocytes at both time points.

Subject	M1				M2			
	Number of *γ*-H2AX foci per 100 lymphocytes (*N*)	Number of *γ*-H2AX foci per affected cell (*N*)	Percentage of affected cells (%)	Diameter of *γ*-H2AX foci (*µ*m)	Number of *γ*-H2AX foci per 100 lymphocytes (*N*)	Number of *γ*-H2AX foci per affected cell (*N*)	Percentage of affected cells (%)	Diameter of *γ*-H2AX foci (*µ*m)
Subject 1	12	1.5	8	0.58	5	1.3	4	0.67
Subject 2	12	1.3	9	0.70	5	1.3	4	0.74
Subject 3	5	1.0	5	0.53	15	1.1	14	0.63
Subject 4	4	1.3	3	0.70	15	1.3	12	0.68
Subject 5	7	1.2	6	0.70	4	1.3	3	0.60
Subject 6	5	1.0	5	0.57	11	1.2	9	0.58
Subject 7	14	1.6	9	0.67	7	1.4	5	0.76
Subject 8	2	1.0	2	0.57	5	1.3	4	0.63

**Table 2:** Data of the four parameters of the *γ*-H2AX foci analysis in human lymphocytes and their test-retest reliability (bias: mean difference between the two measurements; LoA: 95% limits of agreement; CV: coefficient of variation).

*γ*-H2AX foci parameter	M1 (mean ± SD)	M2 (mean ± SD)	Bias	LoA (lower/upper)	CV (%)	*P* value
Number of *γ*-H2AX foci per 100 lymphocytes (*N*)	7.6 ± 4.4	8.4 ± 4.6	0.75	−15.2/13.7	98.5	0.792
Number of *γ*-H2AX foci per affected cell (*N*)	1.2 ± 0.2	1.3 ± 0.1	−0.02	−0.36/0.33	11.3	0.800
Percentage of affected cells (%)	5.9 ± 2.6	6.9 ± 4.2	−1.0	−11.9/9.9	88.9	0.579
Diameter of *γ*-H2AX foci (*µ*m)	0.63 ± 0.07	0.66 ± 0.06	−0.04	−0.16/0.09	8.0	0.334
